# The identification of white matter fibers in the hematoma of intracerebral hemorrhage patients

**DOI:** 10.3389/fncel.2026.1816722

**Published:** 2026-08-06

**Authors:** Jianan Wu, Cheng Zhang, Xian Yu, Huaijun Chen, Haopu Lin, Qizhen He, Chenjun Sun, Guangyu Ying, Feng Cai, Jiaming Xu, Jingsen Chen, Chi Gu, Gao Chen, Jingyin Chen

**Affiliations:** 1 Department of Neurosurgery, Zhejiang University School of Medicine Second Affiliated Hospital, Hangzhou, Zhejiang, China; 2 Zhejiang Key Laboratory of Research and Transformation for Major Neurosurgical Diseases, Hangzhou, Zhejiang, China; 3 ShaoXing Central Hospital, Shaoxing, Zhejiang, China

**Keywords:** fibers, intracerebral hemorrhage, leukocyte infiltration, nerve, white matter

## Abstract

**Background:**

Intracerebral hemorrhage (ICH) carries high mortality, and survivors often have severe neurological deficits. Although surgical clot evacuation is widely used, clinical trials have not demonstrated a clear benefit. White matter is essential for neural network function and may be vulnerable during hematoma evacuation, but whether white matter fibers exist within human ICH hematomas remains unknown. This study aimed to determine the presence and characteristics of intra-hematomal white matter fibers and their relationship with immune cell infiltration.

**Methods:**

We collected hematoma specimens from patients with spontaneous supratentorial ICH undergoing surgical evacuation within 24 h of onset. Histological and immunohistochemical staining, including hematoxylin–eosin and myelin basic protein (MBP), was performed to identify white matter fibers. Lymphatic vessel hyaluronan receptor-1 (LYVE-1) staining was used to evaluate macrophage infiltration around identified fibers.

**Results:**

Myelin-containing structures were consistently observed within the hematoma core on both hematoxylin–eosin and MBP staining. These structures appeared fragmented and structurally fragile. LYVE-1–positive cells were found clustered around intra-hematomal myelin-containing structures, potentially indicating active immune cell infiltration in these regions.

**Conclusion:**

This study provides the first direct histological evidence that white matter fibers persist within human ICH hematomas. This finding provides an anatomical basis for future studies exploring the biological and potential clinical significance of intra-hematomal white matter-associated structures.

## Introduction

Spontaneous intracerebral hemorrhage (ICH) affects four million people worldwide each year (Mendelow et al., 2013). It is the second most common cause of stroke, with high mortality, and few survivors regain functional independence. Mass effect and toxic clot components drive injury after ICH. Till now, whether surgical or conservative treatment is preferable remains controversial. Although removing the hematoma is biologically compelling (Keep et al., 2012), the International Surgical Trial in Intracerebral Hemorrhage trial (STICH), STICH II and the Minimally Invasive Surgery plus rt-PA for Intracerebral Hemorrhage Evacuation trial (MISTIE) did not demonstrate a clear functional benefit of surgery (Mendelow et al., 2005, 2013; Hanley et al., 2019). By contrast, the Early Minimally Invasive Removal of Intracerebral Hemorrhage trial (ENRICH) reported that an early, trans-sulcal parafascicular, tract-aware minimally invasive approach improved 180-day outcomes in selected patients, underlining that how evacuation interacts with white-matter fibers may matter as much as how much blood is removed (Pradilla et al., 2024).

These findings highlight the potential importance of preserving vulnerable neural structures during hematoma evacuation. White matter (WM) fibers are highly vulnerable in ICH and predict the prognosis in different kinds of neurological disorders, serving a critical role in the organization of the distributed neural networks (Novakovic et al., 2021). In humans, more than 77% of ICH patients show white matter injury (WMI) (Smith et al., 2004). Rapid hematoma formation may mechanically compress or disrupt axons, contributing to axonal injury and subsequent degeneration (Puy et al., 2023). Blood-derived products, iron accumulation, oxidative stress, and neuroinflammation can injure oligodendrocytes and oligodendrocyte progenitor cells, resulting in myelin fragmentation and impaired remyelination (Keep et al., 2012). Scores of therapeutic agents and methods were proven effective for the treatment of WMI after ICH in animal models, but without significant success in clinical practice (Zuo et al., 2017). Many WM-targeted interventions succeeded in rodents yet failed clinically, partly because rodents have far less WM than humans and because prior work emphasized peri-hematomal injury (Zuo et al., 2017; Joseph et al., 2016). Our experimental study showed intraclot WM fibers that scaffold microglia or macrophage ingress for erythrocyte clearance in piglets (Chen et al., 2021). In humans, diffusion MRI shows signal consistent with surviving WM within and adjacent to the hematoma (Novakovic et al., 2021). However, such imaging surrogates cannot provide direct evidence of tract-level continuity within the clot, and the presence of intraclot WM fibers in patients remains an indirect inference. Here, we sampled central hematoma tissue from ICH patients undergoing craniotomy to determine the presence of intraclot WM fibers.

## Methods

### Patients and sample collection

We studied a group of 10 patients who underwent craniotomy within 24 h after basal ganglia ICH onset. The ICH diagnosis was made according CT scan. Patients were included based on the following criteria: 1. age ≥ 18 years at randomization; 2. diagnosed as hypertensive basal ganglia intracerebral hemorrhage confirmed by imaging (CT, CTA, etc.); 3. accompanied by functional impairment (such as hematoma-related motor aphasia, sensory aphasia, hemiplegic limb muscle strength ≤ Grade 3 or NIHSS score ≥ 15); 4. the interval from symptom onset to surgery was ≤24 h; 5. pre-stroke mRS score ≤ 1; 6. informed consent obtained in accordance with national laws and regulations and applicable Ethics Committee requirements. The exclusion criteria were as follows: 1. hematoma involving the thalamus, midbrain, ventricles, or other regions; 2. cerebrovascular abnormalities clearly diagnosed by radiological imaging, such as ruptured aneurysm, arteriovenous malformation (AVM), Moyamoya disease, as well as hemorrhagic transformation of ischemic infarction, recent (within 1 year) recurrent intracerebral hemorrhage; 3. any irreversible coagulation disorder or known coagulation system disease; platelet count <100,000/μL; INR > 1.4; use of anticoagulants within 7 days prior to the current ICH; 4. recent possible pregnancy or already pregnant; 5. patients with current severe diseases that may affect outcome assessment.

Age at ICH onset, sex, time between ICH onset and craniotomy, volume of hematoma, whether intraclot white matter fibers were observed, and modified Rankin Scores (mRS) at discharge were collected for all patients. Hematoma volume was estimated on admission CT using the *ABC*/2 method: (*V* = *A* * *B* * *C*/2), where *A* was the greatest hematoma diameter on the axial slice with the largest hemorrhage area, *B* was the greatest diameter perpendicular to *A* on the same slice, and *C* was the number of CT slices containing the hematoma multiplied by the slice thickness. Ten blood clot samples were collected from the center of ICH patients’ intracranial hematoma, respectively, to avoid the influence of peri-hematoma brain tissues. Resections of the hematoma were performed by the senior neurosurgeon, and the routine microsurgical procedure of hematoma clearance was not affected by the sample collection process. This study did not modify current patient care or require the use of further radiological examination and was approved by the medical ethical committees of the Second Affiliated Hospital Zhejiang University School of Medicine (2021-0012).

### Sample histology

The hematoma specimens measuring approximately 5–10 mm in their greatest dimension were fixed in 4% paraformaldehyde (PFA) within 1 h after collection, dehydrated with 30% sucrose solution, embedded in optimal cutting temperature (OCT) compound (SAKURA, OH, USA), and cut into 9-μm-thick sections for the following Hematoxylin–Eosin (H&E) and immunohistochemical staining. For each patient, sections with adequate tissue integrity and staining quality were included in the histological evaluation.

### Hematoxylin and eosin (H&E) staining

H&E staining was performed by Leica ST5010 Autostainer XL. Briefly, slides were rehydrated, stained with hematoxylin and eosin, dehydrated, and sealed with neutral resin.

### Immunohistochemistry

For immunohistochemical staining, slides were immersed in pre-cooled acetone for 10 min, incubated with 3% hydrogen peroxide, blocked with QuickBlotTM Blocking Buffer (Beyotime, Shanghai, China, Cat. No. P0260) for 1 h, and then incubated with primary antibodies including mouse anti-MBP (1:100, Santa Cruz, TX, USA, Cat. No. sc-66064) and rabbit anti-LYVE-1(1:5000, Abcam, Cambridge, UK, Cat. No. ab-219556) at 4 °C overnight. Slides were incubated with secondary antibody for 1 h and then stained with Diaminobenzidine (DAB) for 2 min to visualize immune complexes. The secondary antibody and DAB staining system were purchased from genetech (Shanghai, China, Cat. No. GK6005).

### Statistical analysis

This was an exploratory descriptive histopathological study primarily designed to determine whether white matter fibers could be identified within the hematoma. No formal inferential statistical analysis was performed. Clinical and histological findings were summarized descriptively.

## Results

**Figure 1:**
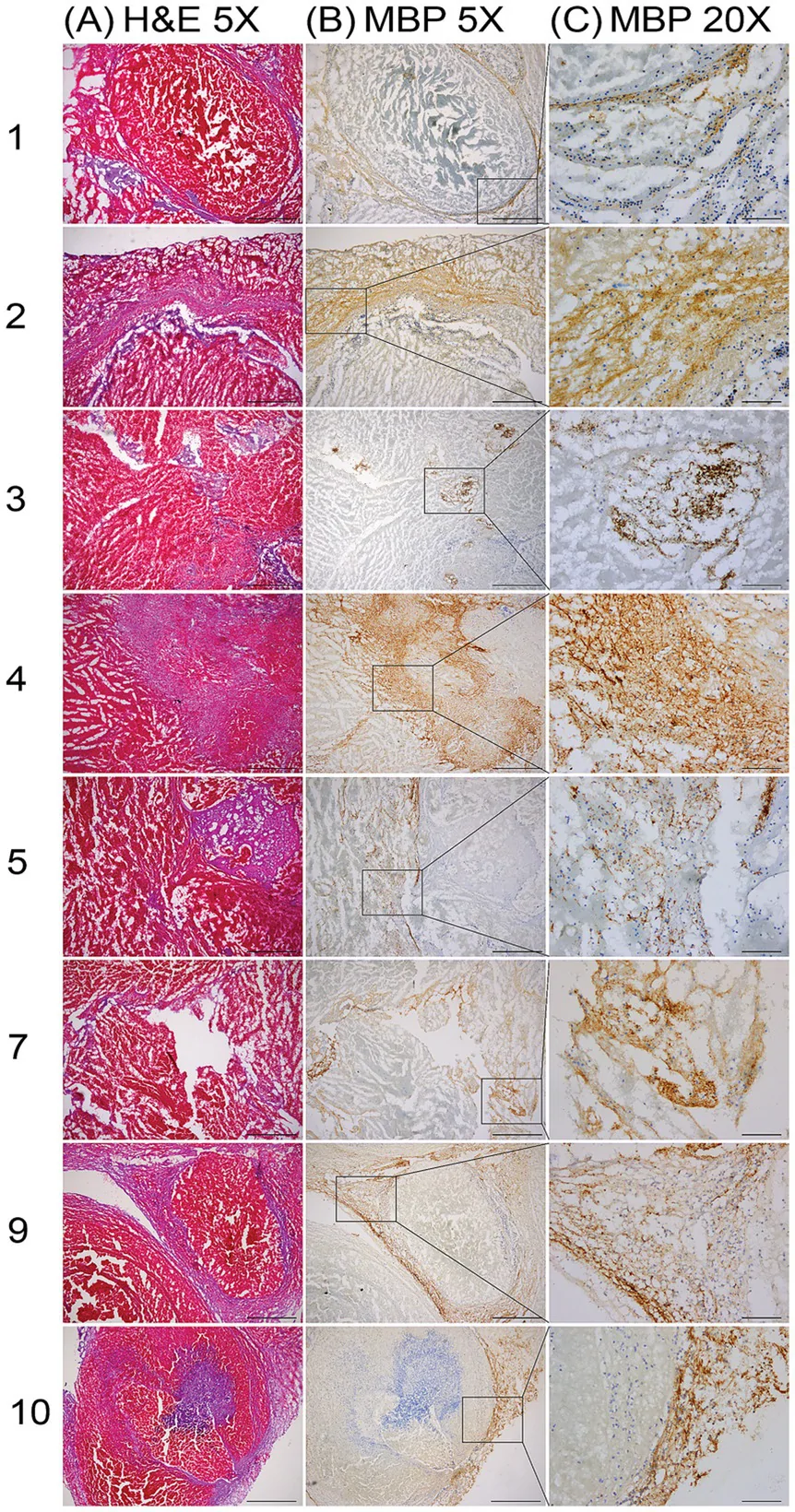
White matter fibers were identified within the hematoma of ICH patients. Row **(A)** H&E staining of white matter fibers inside the hematoma of ICH patients. Scale bar = 500 μm. Row **(B,C)** MBP staining of white matter fibers inside the hematoma in both low (row **B**) and high (row **C**) magnification. Scale bar in row **(B)** = 500 μm; Scale bar in row **(C)** = 100 μm.

We collected hematoma from 10 patients during craniotomy within 24 h after ICH onset. Age at ICH onset, sex, time between ICH onset and craniotomy, volume of hematoma, whether intraclot white matter fibers were observed, and modified Rankin Scores (mRS) at discharge were collected for all patients. Characteristics of patients are listed in Table 1. Among the 10 samples, intra-hematomal white matter fibers were observed in 8 hematoma clots (Table 1). The identification of white matter fibers in the hematoma of ICH patients was shown by H&E staining (Figure 1, row A). To further confirm the presence of white matter fibers in the hematoma, myelin basic protein (MBP, one of the major constituents of myelin) immunohistochemistry was performed. MBP-positive structures were detected within the hematoma (Figure 1, rows B,C), indicating the presence of myelin-containing tissue.

**Table 1:** Characteristics of patients.

No.	Age	Gender	Time between ICH onset and craniotomy (h)	Volume of hematoma (mL)	Intraclot white matter fibers observed (yes/no)	mRS
1	34	M	4	63	Yes	4
2	66	F	4	93	Yes	5
3	60	M	13	129	Yes	5
4	63	M	12	122	Yes	4
5	70	M	12	82	Yes	5
6	37	M	7	65	No	5
7	63	M	12	49	Yes	4
8	80	M	20	48	No	4
9	44	M	24	66	Yes	5
10	37	M	7	65	Yes	5

**Figure 2:**
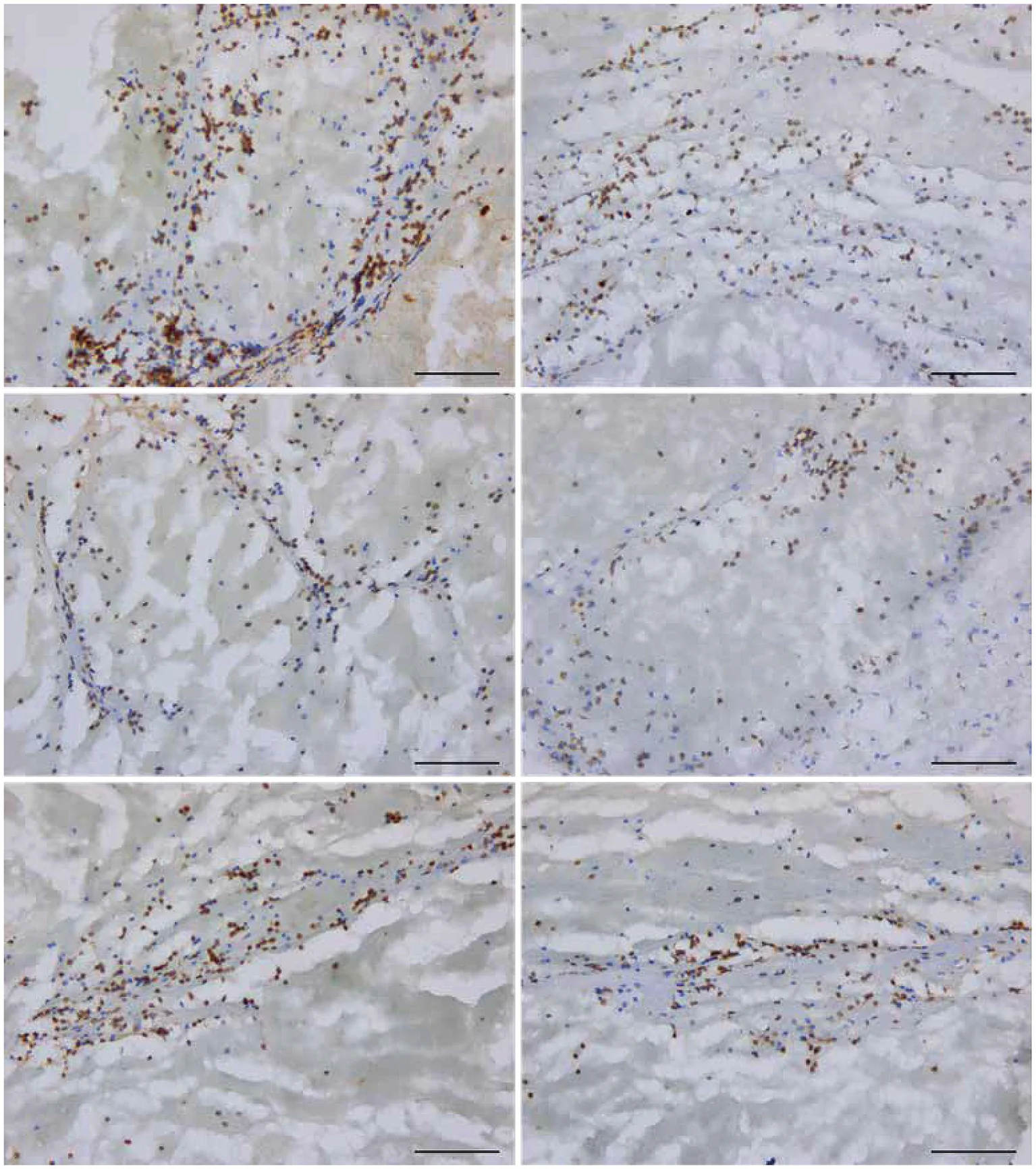
LYVE-1-positive cells were centered around the white matter fibers. Distribution of LYVE-1 positive cells in the hematoma with white matter fibers. Scale bar = 100 μm.

Beyond being a lymphatic marker, lymphatic vessel hyaluronan receptor-1 (LYVE-1) is expressed on subsets of infiltrating or activated macrophages, structurally related to CD44 and other hyaluronan-binding proteins (Brezovakova and Jadhav, 2020). We therefore used LYVE-1 to identify microglia/macrophage-associated cells within the intraclot white matter. Most of the LYVE-1 positive cells were centered around the white matter fibers (Figure 2). The major findings of this study include: (1) myelin-containing structures were identified in the hematoma core within the first 24 h in ICH patients; (2) LYVE-1 positive cells (microglia/macrophages) showed a spatial association with intraclot white matter fibers.

## Discussion

Previous research showed the absence of white matter fibers within the hematoma and mainly focused on injury in the peri-hematomal region in rodent ICH animal models. Our recent study, via the porcine model, reveals that morphologically normal white matter fibers are present within the hematoma core post-ICH and that these fibers survive even after the hematoma resolves at 2 months after ICH (Chen et al., 2021). To our knowledge, this is the first study to provide direct histological evidence of intraclot white matter fibers in human hematoma specimens obtained within 24 h after ICH onset.

Microglia/macrophages, serving an important role in preserving tissue integrity and function by engulfing old and damaged cells, are key phagocytes engaged in hematoma clearance (Chang et al., 2018; Hemphill et al., 2015). In our study, we found numerous LYVE-1-positive cells infiltrated into the intraclot white matter fibers. In the present study, meningeal lymphatic vessels were anatomically distant from the basal ganglia hematoma. Moreover, LYVE-1 staining was observed in discrete, individually distributed cells, without a continuous endothelial lining or well-defined luminal structures. Taken together, these findings suggest that the LYVE-1-positive cells were more likely to belong to the microglial/macrophage lineage. In addition, proinflammatory cytokines induce uptake and degradation of LYVE-1, resulting in a reduction of LYVE-1 receptors *in vitro*. These studies indicate the LYVE-1 positive cells we observed in the intraclot white matter fibers may be related to the degradation of blood components and be associated with the blood clearance process after stroke.

Intervention of spontaneous ICH for most patients still remains controversial. Most randomized trials comparing surgical intervention with conservative treatment, including STICH (Mendelow et al., 2005), MISTIE III (Hanley et al., 2019) and the Minimally Invasive Surgery vs. Medical Management Alone for Intracerebral Hemorrhage trial (MIND) (Arthur et al., 2025), have not demonstrated an overall functional benefit (Jackson, 2004).

Conversely, ENRICH demonstrated benefit that depended on hemorrhage location and the surgical pathway, using an early trans-sulcal approach guided by white matter tracts (Pradilla et al., 2024). Complementary cohort data indicate that image-guided para-corticospinal tract (CST) corridors are associated with higher 90-day independence and that postoperative volume does not fully explain outcome, pointing to the importance of corridor–white matter interactions, and peri-operative tractography further demonstrates the feasibility of CST-sparing minimally invasive surgery (Soto et al., 2025; Zhang et al., 2021). White matter contains the major axonal pathways that connect different brain regions and support neural signal transmission. In the present study, MBP-positive structures within the hematoma may reflect pre-existing myelinated tissue that was incorporated into the clot during hematoma expansion rather than being immediately destroyed. Their detection within 24 h suggests that myelin-associated components may persist during the acute phase of ICH. However, during the surgical and invasive approach, in order to reach the hematoma that usually takes deep brain structures, a large layer of healthy cerebral tissue needs to be dissected, and intra-hematomal white matter fibers may be disrupted (de Oliveira Manoel, 2020). In fact, even with a minimally invasive catheter evacuation approach, the white matter fibers inside the hematoma observed in our current study are vulnerable and easy to be evacuated together with the hematoma. These may impact the effects of these white matter fibers on trafficking cellular signals, regeneration, and clot clearance, whereas intact brain tissue and white matter fibers overlying and within the blood clot could contribute to axon regeneration and hematoma clearance.

This study suggests that white matter fibers can be identified within the hematoma of ICH patients and that these intraclot white matter fibers are related to microglia/macrophages infiltrating, which may play a role in hematoma clearance. In our previous porcine study, regions of the hematoma containing intraclot white matter fibers showed significantly higher numbers of heme oxygenase-1 (HO-1)-positive and macrophage scavenger receptor 1 (MSR1)-positive microglia/macrophage-like cells than regions without white matter fibers. These cells were also spatially concentrated around the fibers, suggesting a close association between intraclot white matter and phagocyte infiltration. These findings support the possibility that intraclot white matter fibers may provide a structural scaffold or route for immune-cell infiltration and potentially contribute to hematoma clearance (Chen et al., 2021). Although the present human study does not directly establish this mechanism, the similar spatial relationship observed in surgical specimens provides preliminary support for its possible relevance in patients. Further clinical and mechanistic studies are needed to determine whether preserving intraclot white matter fibers improves neurological outcomes and to develop more targeted hematoma evacuation strategies that minimize unnecessary disruption of these structures.

This study has several limitations. First, the sample size was small and all specimens were obtained from patients who underwent craniotomy, which may introduce selection bias and limit the generalizability of the findings. Second, the proportion and spatial distribution of myelin-containing structures within the entire hematoma have not been reliably quantified. Third, MBP positivity indicates the presence of myelin-associated components but cannot confirm functional viability. Future studies incorporating markers of axonal integrity, such as neurofilament or SMI312, are needed to further characterize these structures. Fourth, LYVE-1 staining alone cannot definitively establish that the positive cells belong to the microglial/macrophage cells. Future studies should include co-staining with more established myeloid markers, such as Iba1, CD68, or CD163, to further determine their cellular identity. Finally, the observed association between LYVE-1-positive cells and myelin-containing structures was based on spatial colocalization and does not establish a causal role in hematoma clearance.

## Conclusion

In conclusion, we demonstrate that white matter fibers can be detected within the hematoma core of spontaneous basal ganglia ICH patients undergoing craniotomy within 24 h of onset. Using H&E staining and MBP immunohistochemistry, intraclot myelinated fibers were observed in 8 of 10 clot samples, indicating that tract-like structures may persist inside the clot rather than being restricted to the peri-hematomal region. In addition, LYVE-1–positive microglia/macrophages preferentially clustered around these fibers, suggesting that intraclot white matter may provide a structural substrate for immune cell infiltration and erythrocyte clearance. Our findings provide a preliminary anatomical rationale for tract-aware hematoma evacuation strategies that minimize disruption of intraclot white matter, which warrant further investigation in future studies.

## Data Availability

The raw data supporting the conclusions of this article will be made available by the authors, without undue reservation.
